# Previous Use of Mammography as a Proxy for General Health Checks in Association with Better Outcomes after Major Surgeries

**DOI:** 10.3390/ijerph16224432

**Published:** 2019-11-12

**Authors:** Ying-Hsuan Tai, Ta-Liang Chen, Yih-Giun Cherng, Chun-Chieh Yeh, Chuen-Chau Chang, Chien-Chang Liao

**Affiliations:** 1 Department of Anesthesiology, Shuang Ho Hospital, Taipei Medical University, New Taipei City 235, Taiwan; tp16960@gmail.com (Y.-H.T.); stainless@s.tmu.edu.tw (Y.-G.C.); 2 Department of Anesthesiology, School of Medicine, College of Medicine, Taipei Medical University, Taipei 110, Taiwan; tlc@tmu.edu.tw (T.-L.C.); nekota@tmu.edu.tw (C.-C.C.); 3 Department of Anesthesiology, Wan Fang Hospital, Taipei Medical University, Taipei 116, Taiwan; 4 Department of Surgery, China Medical University Hospital, Taichung 404, Taiwan; b8202034@gmail.com; 5 Department of Surgery, University of Illinois, Chicago, IL 60607, USA; 6 Department of Anesthesiology, Taipei Medical University Hospital, Taipei 110, Taiwan; 7 Anesthesiology and Health Policy Research Center, Taipei Medical University Hospital, Taipei 110, Taiwan; 8 Research Center of Big Data and Meta-Analysis, Wan Fang Hospital, Taipei Medical University, Taipei 116, Taiwan; 9 School of Chinese Medicine, College of Chinese Medicine, China Medical University, Taichung 404, Taiwan

**Keywords:** mammography, surgery, complications, mortality

## Abstract

Although previous studies have shown that health checks may improve several risk factors for chronic diseases, the effect of preoperative health checks on postoperative recovery in surgical patients remains unknown. We aimed to investigate the association between preoperative use of mammography and the risk of perioperative complications. We conducted a matched cohort study of 152,411 patients aged ≥47 years who received mammography screening and later underwent major surgery from 2008 to 2013. Using a propensity score matching procedure adjusted for sociodemographic characteristics, medical condition, surgery type, and anesthesia type, 152,411 controls who underwent surgery but were not screened were selected. We collected patients’ characteristics and medical conditions from claims data of Taiwan’s National Health Insurance. Logistic regressions were used to calculate odds ratios (ORs) with 95% confidence intervals (CIs) for postoperative complications and in-hospital mortality associated with mammography screening. Patients receiving mammography prior to major surgery had significantly lower risks of perioperative complications, including pneumonia, septicemia, acute renal failure, stroke, urinary tract infection, deep wound infection, acute myocardial infarction, intensive care unit stay, and 30 day in-hospital mortality (OR 0.45, 95% CI 0.38–0.53). The association was consistent across each stratum of age, number of hospitalizations, emergency visits, and comorbidities. In conclusion, preoperative use of mammography was strongly associated with fewer perioperative complications and less in-hospital mortality after major surgeries. The evidence provided by this study justifies the implementation of preoperative health checks in clinical practice.

## 1. Introduction

Over 300 million surgical procedures were performed worldwide in 2012, and the global volume of surgery continues to increase [1]. Multiple factors influence the postoperative outcome, including patient factors, the clinical experience of surgeons, and the healthcare quality of hospitals [2,3]. Regarding patient factors, patients’ underlying health conditions and knowledge, attitude, and practices about diseases significantly affect treatment effectiveness and clinical outcome [4]. To enhance the postoperative outcome, it is important for clinicians to optimize patients’ health conditions and improve their health awareness before surgery.

In preventive medicine, the aim of health checks is to prevent the development or progression of a disease by conducting comprehensive examinations that identify and manage chronic diseases and their risk factors, most of which are cardiovascular diseases or malignancies [5]. It is postulated that early detection and management of biomedical and lifestyle risk factors are able to avert or delay chronic diseases. Current studies have demonstrated that periodic health evaluations can reduce established risk factors of chronic diseases and improve the delivery of some recommended preventive services [5,6]. Nonetheless, it is currently unknown whether health checks prior to surgery improve patients’ postoperative recovery.

Mammography has been one of the comprehensive examinations in the national health check program under the coverage of national health insurance in Taiwan [7]. Women aged between 45 and 69 years or those aged between 40 and 44 years with a family history of breast cancer are offered free mammograms biennially [8]. To investigate the putative beneficial effect of preoperative health checks on short-term outcomes in patients following major surgeries, mammography was used as a representative of health checks in this nationwide cohort study. We hypothesized that patients receiving preoperative mammography had a lower risk of major complications and in-hospital mortality following major surgery compared to their counterparts.

## 2. Methods

### 2.1. Source of Data

The reimbursement claims data of Taiwan’s National Health Insurance was used in this study. The insurance program was established in March 1995 and covers more than 99% of all Taiwanese residents. The research database of Taiwan’s National Health Insurance includes inpatient and outpatient demographic characteristics, physicians’ primary and secondary diagnoses, treatment procedures, prescriptions, and medical expenditures. Research articles based on data from this database have been accepted in outstanding scientific journals [9,10]. For the purpose of protecting personal privacy, the electronic database was decoded, with patient identifications scrambled for further public access for research. This study was conducted in accordance with the Helsinki Declaration. According to official regulations, informed consent is not required because of the use of decoded and scrambled patient identifications. However, this study was evaluated and approved by the Institutional Review Board of Taipei Medical University (TMU-JIRB-201905042; TMU-JIRB-201902053). 

### 2.2. Study Design

**Figure 1:**
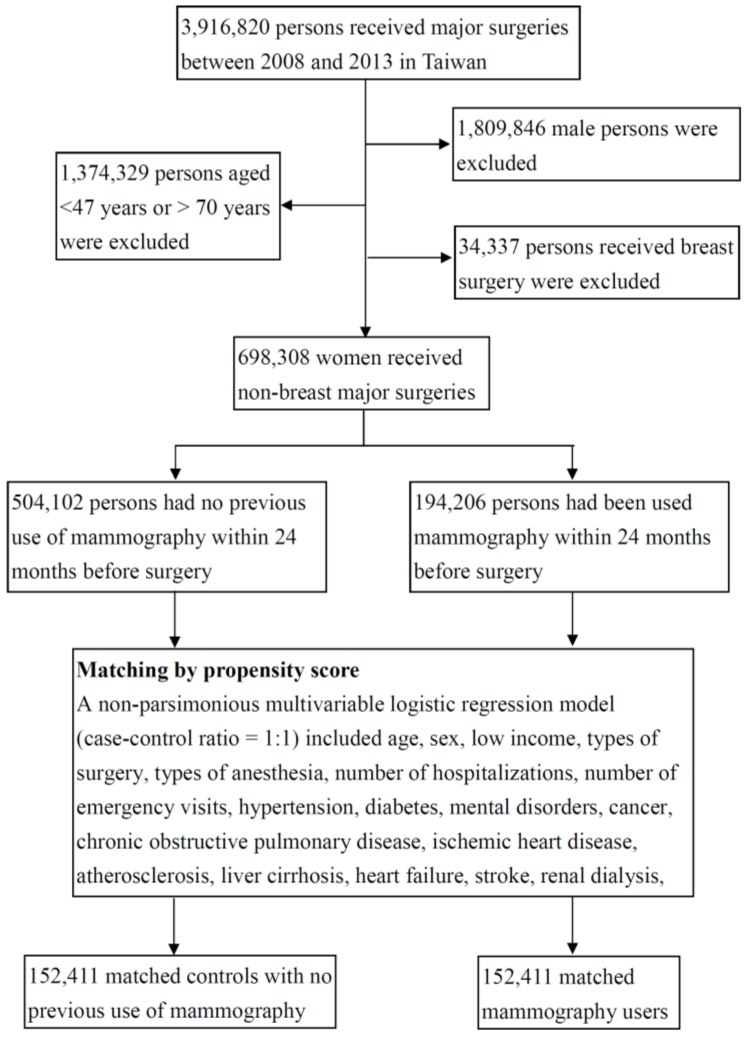
The selection process of study subjects.

Among the 3.9 million Taiwan residents who underwent major inpatient surgeries (defined as procedures requiring general, epidural, or spinal anesthesia, as well as hospitalization for >1 day) from 2008 to 2013, we examined medical claims and identified 698,308 female patients aged 47–70 years (Supplementary Materials, Table S1). Of these, 194,206 had received a mammography exam within 24 months before the index surgery. Each patient who underwent surgery and received mammography was randomly matched to a patient without mammography who underwent surgery using a propensity score matched-pair procedure. After propensity score matching (case-control ratio, 1:1), there were 152,411 patients with and 152,411 without preoperative mammography (Figure 1).

### 2.3. Measures and Definitions

By the regulations of the Bureau of National Health Insurance in Taiwan, people with low-income status were qualified to have the registration fee and medical copayment waived when visiting medical services. Low-income status in this study was defined as having a low income within two years before surgery. The number of hospitalizations and emergency visits of patients who visited medical services for inpatient care or emergency care within 24 months before the index surgery were identified. According to the International Classification of Diseases, Ninth Revision, Clinical Modification (ICD-9-CM) and physicians’ diagnoses, we defined patients’ medical conditions including hypertension (ICD-9-CM 401–405), diabetes (ICD-9-CM 250), mental disorders (ICD-9-CM 290–319), cancer (ICD-9-CM 140-208, 230–234), chronic obstructive pulmonary disease (ICD-9- CM 491, 492, 496), ischemic heart disease (ICD-9-CM 410–414), atherosclerosis (ICD-9-CM 440), liver cirrhosis (ICD-9-CM 571.2, 571.5, 571.6), heart failure (ICD-9-CM 428), stroke (ICD-9-CM 430–438), and Parkinson’s disease (ICD-9-CM 332). Renal dialysis was defined by administration codes (D8 and D9). These coexisting medical conditions were identified 24 months before the index surgery. Postoperative complications within 30 days after surgery included postoperative bleeding, deep wound infection, stroke, pneumonia, septicemia, urinary tract infection, pulmonary embolism, and acute renal failure and were also defined according to ICD-9-CM and physician’s diagnosis. In this study, the medical expenditures of surgical patients were calculated by counting all National Health Insurance payments during the index surgical hospitalization. This included payments for surgical procedures, medications, admission stay, and materials. The length of hospital stay was calculated from the first day to the last day of surgical hospitalization.

### 2.4. Statistical Analysis

We used matched analysis with propensity score to determine associations between mammography and postoperative outcomes. A nonparsimonious multivariable logistic regression model was used to estimate a propensity score for surgical patients who did or did not receive mammography. Clinical significance guided the initial choice of covariates in this logistic model to include age, sex, low income, hypertension, diabetes, mental disorders, cancer, chronic obstructive pulmonary disease, ischemic heart disease, atherosclerosis, liver cirrhosis, heart failure, stroke, Parkinson’s disease, and renal dialysis. We matched mammography recipients to nonrecipients using a greedy matching algorithm (without replacement) with a caliper width of 0.2 SDs of the log odds of the estimated propensity score. Categorical variables are summarized using frequencies (%) and were compared between mammography recipients and nonrecipients by using chi-square tests. Continuous variables are presented as the means ± standard deviations and were compared using t-tests. Logistic regressions were used to calculate the adjusted odds ratios (ORs) and 95% confidence intervals (CIs) of postoperative adverse outcomes associated with mammography. We also performed further analyses stratified by age, number of hospitalizations, number of emergency visits, and number of medical conditions to examine the association between mammography and postoperative adverse events within these strata.

## 3. Results

Table 1 shows the baseline characteristics of patients with and without mammography who underwent major surgeries. Because we used matched analysis with a propensity score, there were no significant differences between the groups of surgical patients with and without mammography analyzed by age, low income, types of surgery and anesthesia, number of hospitalizations, number of emergency visits, hypertension, diabetes, mental disorders, cancer, chronic obstructive pulmonary disease, ischemic heart disease, atherosclerosis, liver cirrhosis, heart failure, stroke, Parkinson’s disease, and renal dialysis.

Compared with patients without mammography, patients with mammography had lower risks of postoperative stroke (OR 0.56, 95% CI 0.53–0.60), acute myocardial infarction (OR 0.62, 95% CI 0.48–0.80), acute renal failure (OR 0.54, 95% CI 0.47–0.62), deep wound infection (OR 0.85, 95% CI 0.76–0.96), septicemia (OR 0.75, 95% CI 0.71–0.78), pneumonia (OR 0.64, 95% CI 0.59–0.70), urinary tract infection (OR 0.93, 95% CI 0.90–0.96), and in-hospital mortality (OR 0.45, 95% CI 0.38–0.53) after surgery (Table 2). Mammography was also associated with admission to the intensive care unit after surgery (OR 0.68, 95% CI 0.66–0.70). The mean amount of medical expenditures ($2392 ± $2627 vs. $2579 ± $3046, *p* < 0.0001) and length of hospital stay (6.0 ± 6.5 vs. 6.9 ± 8.3 days, *p* < 0.0001) were significantly lower for patients with mammography than for patients without mammography.

In the stratification analysis (Table 3), associations between mammography and reduced postoperative adverse events were significant in women aged 47–49 years (OR 0.8, 95% CI 0.71–0.90), 50–54 years (OR 0.66, 95% CI 0.61–0.71), 55–59 years (OR 0.7, 95% CI 0.65–0.75), 60–64 years (OR 0.65, 95% CI 0.60–0.70), and 65–70 years (OR 0.64, 95% CI 0.59–0.69). Use of mammography was also associated with reduced postoperative adverse events in women who had 0 (OR 0.62, 95% CI 0.58–0.65), 1 (OR 0.69, 95% CI 0.66–0.73), 2 (OR 0.73, 95% CI 0.67–0.79), 3 (OR 0.73, 95% CI 0.62–0.85), or ≥4 (OR 0.64, 95% CI 0.44–0.93) medical conditions. The reduced risk of postoperative adverse events was associated with use of mammography in women and the number of emergency visits (0, 1, 2, ≥3) and hospitalizations (0, 1, 2, ≥3). Compared with the control group (Table 4), the adjusted ORs of postoperative adverse events associated with use of mammography within preoperative 30, 60, 90, 120, and 365 days were 0.64 (95% CI 0.57–0.71), 0.65 (95% CI 0.60–0.70), 0.65 (95% CI 0.60–0.69), 0.65 (95% CI 0.61–0.69), and 0.65 (95% CI 0.63–0.68), respectively.

## 4. Discussion

With large-scale, population-based, and cross-sectional analyses adjusted for patients’ demographics, hospital characteristics, medical comorbidities, and types of surgeries, we found that surgical patients with preoperative use of mammography had significantly lower postoperative complications and mortality. Using mammography preoperatively was associated with a reduced risk of intensive care, shorter length of hospital stay, and lower medical expenditures. The association between preoperative use of mammography and postoperative adverse events was significant in many various subgroups. Our study is the first to investigate the preoperative use of mammography as a representative of health checks that are associated with better postoperative outcomes.

Whether general health checks decrease morbidity and mortality from diseases remains unclear, and the results of previous studies are mixed and inconclusive. Health checks have been reported to improve the detection and control of diseases and their risk factors [5,6,11,12]. However, a meta-analysis including 14 randomized trials demonstrated that general health checks did not reduce morbidity or mortality caused by either cardiovascular disease or cancer [13]. Despite these analyses, there is a lack of evidence about the effect of health checks on surgical patients. Our study demonstrated how mammography is beneficial for postoperative outcomes, which may be explained by the following four reasons.

First, screening mammography was typically combined with other examinations for common chronic diseases in the Taiwanese national health check program, including fecal immunochemical tests for colorectal cancer, pap smear tests for cervical cancer, blood pressure, and fasting blood glucose measurement [7]. Taiwanese health check programs have been suggested to reduce the mortality rates in breast cancer [8], colorectal cancer [14], and hepatocellular carcinoma [15]. In our study, patients receiving mammography had a lower mortality risk, which may be attributed to such health checks identifying and treating previously unrecognized diseases or improving the control of pre-existing medical conditions. Various comorbidities have been reported to adversely affect postoperative recovery. For example, hypertension, poor preoperative glycemic control, and morbid obesity are established risk factors of perioperative cardiovascular events and mortality [16,17,18,19]. Health checks have been shown to enhance the control of blood pressure and body mass index and decrease modeled cardiovascular disease risk [5,11].

Second, those not attending screening programs were more likely to have limited literacy skills and health knowledge [20,21]. One national survey indicated that approximately 30% of Taiwanese adults have low health literacy [22], and low literacy is linked to several adverse health outcomes, including health knowledge, intermediate disease markers, measures of morbidity, general health condition, and utilization of health resources [23]. In addition, patients’ health knowledge and attitude may have a practical effect on self-care behavior and medical compliance in chronic illnesses, such as hypertension and diabetes [4,24]. Educational interventions in health checks may improve health knowledge and medication adherence in patients with low health literacy levels [25]. In Taiwanese health check programs, participants would be given instruction and education to increase their knowledge of common chronic diseases and to influence their perception and attitude towards them [7].

Third, nonparticipants in mammography screening may have lower social support for medical care, smaller social network size, and less outpatient utilization compared with participants, which may impede their access and adherence to health check programs [26]. Patients’ social support may significantly affect postoperative recovery and is predictive of surgical outcome, including the length of hospital stay and mortality [27,28,29]. In addition, patients’ social support and social network members have an important influence on chronic illness self-management, which may further affect postoperative recovery and the risk of complications [30].

Fourth, research has shown that low-income women are less likely to obtain mammograms than their more affluent counterparts regardless of free services [31], and each $10,000 increment in annual household income increased mammography utilization rates by 2.5% [32]. Low socioeconomic status is associated with increased operative and cardiovascular mortality rates among surgical patients [33,34]. Of note, our analysis has accounted for patients’ income to evaluate the independent association between the use of mammography and surgical outcome.

Attention to some limitations of this study is needed. First, our study is an observational study without randomized selection of exposure and nonexposure groups. The effects of unmeasured confounders cannot be fully adjusted, and self-selection bias cannot be avoided. Second, we have no clinical data on detailed surgical and anesthetic techniques, physicians’ personal skills and experiences, and some medical treatment materials that were not covered by health insurance. We also have no information regarding the family history of breast cancer in this study. Third, the analysis cannot distinguish between organized screening and opportunistic screening in which the mammography was performed. Patients undergoing mammography in opportunistic screening may not have additional health evaluations performed in multiple screening programs and, therefore, have a lower chance to detect and treat comorbid diseases. Fourth, information on education level, job, unemployment, and family status (living alone or with families) was not available in Taiwan’s National Health Insurance Research Database. This is also a study limitation, because we could not evaluate these factors on the association between mammography use and postoperative outcomes. In addition, information regarding smoking, drinking alcohol, physical activity, and other lifestyle characteristics was not available in the insurance claims. Finally, our study focuses on female surgical populations, which limited the generalizability of the findings.

## 5. Conclusions

In conclusion, previous use of mammography as a proxy of health checks was associated with reduced postoperative complications, in-hospital mortality, and consumption of medical resources. The beneficial effect was consistent across each stratum of age, number of hospitalizations, emergency visits, and comorbidities. Our results did not implicate a causal relationship between use of mammography and postoperative outcome. Further studies are warranted to investigate whether the promotion of health behaviors has perioperative outcome benefits.

## Figures and Tables

**Table 1:** Characteristics of surgical patients with and without preoperative use of mammography.

Characteristics	Use of Mammography				*p*-Value
	No (*n* = 152,411)		Yes (*n* = 152,411)		
**Age, years**	***n***	**(%)**	***n***	**(%)**	**1.0000**
47–49	17,450	(11.5)	17,450	(11.5)	
50–54	38,469	(25.2)	38,469	(25.2)	
55–59	37,676	(24.7)	37,676	(24.7)	
60–64	32,143	(21.1)	32,143	(21.1)	
65–70	26,673	(17.5)	26,673	(17.5)	
Low income					1.0000
No	15,1396	(99.3)	15,1396	(99.3)	
Yes	1015	(0.7)	1015	(0.7)	
Number of hospitalizations					1.0000
0	12,2469	(80.4)	12,2469	(80.4)	
1	22,834	(15.0)	22,834	(15.0)	
2	4017	(2.6)	4017	(2.6)	
≥3	3091	(2.0)	3091	(2.0)	
Number of emergency visits					1.0000
0	108,688	(71.3)	108,688	(71.3)	
1	30,014	(19.7)	30,014	(19.7)	
2	8579	(5.6)	8579	(5.6)	
≥3	5130	(3.4)	5130	(3.4)	
Types of surgery					1.0000
Musculoskeletal	52,827	(34.7)	52,827	(34.7)	
Digestive	24,466	(16.1)	24,466	(16.1)	
Neurosurgery	19,619	(12.9)	19,619	(12.9)	
Kidney, ureter, bladder	9539	(6.3)	9539	(6.3)	
Respiratory	5462	(3.6)	5462	(3.6)	
Cardiovascular	2620	(1.7)	2620	(1.7)	
Eye	1657	(1.1)	1657	(1.1)	
Skin	1494	(1.0)	1494	(1.0)	
Delivery, cesarean section, abortion	150	(0.1)	150	(0.1)	
Others	34,577	(22.7)	34,577	(22.7)	
Types of anesthesia					1.0000
General	124,417	(81.6)	124,417	(81.6)	
Epidural or spinal	27,994	(18.4)	27,994	(18.4)	
Medical conditions					
Hypertension	40,639	(26.7)	40,639	(26.7)	1.0000
Diabetes	19,838	(13.0)	19,838	(13.0)	1.0000
Mental disorders	25,142	(16.5)	25,142	(16.5)	1.0000
Cancer	19,580	(12.9)	19,580	(12.9)	1.0000
COPD	10,318	(6.8)	10,318	(6.8)	1.0000
Ischemic heart disease	6964	(4.6)	6964	(4.6)	1.0000
Atherosclerosis	1997	(1.3)	1997	(1.3)	1.0000
Liver cirrhosis	1936	(1.3)	1936	(1.3)	1.0000
Heart failure	578	(0.4)	578	(0.4)	1.0000
Stroke	1059	(0.7)	1059	(0.7)	1.0000
Renal dialysis	684	(0.5)	684	(0.5)	1.0000
Parkinson’s disease	326	(0.2)	326	(0.2)	1.0000

COPD, chronic obstructive pulmonary disease.

**Table 2:** Outcomes after major surgeries in patients with and without mammography.

Postoperative Outcomes	Use of Mammography				Risk of Outcomes	
	No (*n* = 152,411)		Yes (*n* = 152,411)			
	Events	%	Event	%	OR	(95% CI)*
30 day in-hospital mortality	466	0.3	212	0.1	0.45	(0.38–0.53)
Postoperative complications						
Pneumonia	1378	0.9	895	0.6	0.64	(0.59–0.70)
Septicemia	3879	2.6	2922	1.9	0.75	(0.71–0.78)
Acute renal failure	534	0.4	291	0.2	0.54	(0.47–0.62)
Pulmonary embolism	106	0.1	98	0.1	0.92	(0.70–1.22)
Stroke	2660	1.8	1542	1.0	0.56	(0.53–0.60)
Urinary tract infection	6654	4.4	6233	4.1	0.93	(0.90–0.96)
Deep wound infection	645	0.4	550	0.4	0.85	(0.76–0.96)
Acute myocardial infarction	158	0.1	99	0.1	0.62	(0.48–0.80)
Postoperative bleeding	699	0.5	695	0.5	0.99	(0.90–1.11)
Intensive care unit stay	10,361	6.8	7506	4.9	0.68	(0.66–0.70)
Medical expenditure, USD†	2579 ± 3046		2392 ± 2627		*p* < 0.0001	
Length of hospital stay, days†	6.9 ± 8.3		6.0 ± 6.5		*p* < 0.0001	

CI, confidence interval; OR, odds ratio; *Adjusted for all covariates listed in Table 1; † Mean ± SD.

**Table 3:** The stratified analysis for the association between use of mammography and postoperative adverse events.

Factors of Stratification			Adverse Events*			
Stratums	Mammography	*n*	Events	Rate, %	OR	(95% CI)†
Age 47–49 years	No	17,450	627	3.6	1.00	(reference)
	Yes	17,450	504	2.9	0.80	(0.71–0.90)
Age 50–54 years	No	38,469	1644	4.3	1.00	(reference)
	Yes	38,469	1109	2.9	0.66	(0.61–0.71)
Age 55–59 years	No	37,676	1819	4.8	1.00	(reference)
	Yes	37,676	1304	3.5	0.70	(0.65–0.75)
Age 60–64 years	No	32,143	1869	5.8	1.00	(reference)
	Yes	32,143	1250	3.9	0.65	(0.60–0.70)
Age 65–70 years	No	26,673	1855	7.0	1.00	(reference)
	Yes	26,673	1233	4.6	0.64	(0.59–0.69)
0 hospitalization	No	122,469	5798	4.7	1.00	(reference)
	Yes	122,469	3890	3.2	0.65	(0.63–0.68)
1 hospitalization	No	22,834	1302	5.7	1.00	(reference)
	Yes	22,834	940	4.1	0.71	(0.65–0.77)
2 hospitalization	No	4017	331	8.2	1.00	(reference)
	Yes	4017	271	6.8	0.80	(0.68–0.95)
≥3 hospitalizations	No	3091	383	12.4	1.00	(reference)
	Yes	3091	299	9.7	0.75	(0.63–0.88)
0 emergency visits	No	108,688	5003	4.6	1.00	(reference)
	Yes	108,688	3292	3.0	0.64	(0.61–0.67)
1 emergency visit	No	30,014	1685	5.6	1.00	(reference)
	Yes	30,014	1237	4.1	0.72	(0.67–0.77)
2 emergency visits	No	8579	611	7.1	1.00	(reference)
	Yes	8579	467	5.4	0.75	(0.66–0.85)
≥3 emergency visits	No	5130	515	10.0	1.00	(reference)
	Yes	5130	404	7.9	0.76	(0.66–0.87)
0 medical condition	No	61,938	2723	4.4	1.00	(reference)
	Yes	61,938	1720	2.8	0.62	(0.58–0.65)
1 medical condition	No	60,228	3184	5.3	1.00	(reference)
	Yes	60,228	2266	3.8	0.69	(0.66–0.73)
2 medical conditions	No	23,692	1462	6.2	1.00	(reference)
	Yes	23,692	1087	4.6	0.73	(0.67–0.79)
3 medical conditions	No	5553	368	6.6	1.00	(reference)
	Yes	5553	275	5.0	0.73	(0.62–0.85)
≥4 medical conditions	No	1000	77	7.7	1.00	(reference)
	Yes	1000	52	5.2	0.64	(0.44–0.93)

CI, confidence interval; OR, odds ratio; *Adverse events included with 30 day in-hospital mortality, pneumonia, septicemia, acute renal failure, stroke, and acute myocardial infarction; †Adjusted for all covariates listed in Table 1.

**Table 4:** The stratified analysis for the association between use of mammography and postoperative adverse events.

Time Period Before Surgery		Adverse Events*			
	*n*	Events	Rate, %	OR	(95% CI)†
No use of mammography	152,411	7814	5.1	1.00	(reference)
Use mammography within 30 days	13,184	387	2.9	0.64	(0.57–0.71)
Use mammography within 60 days	22,903	705	3.1	0.65	(0.60–0.70)
Use mammography within 90 days	30,556	961	3.2	0.65	(0.60–0.69)
Use mammography within 120 days	37,492	1199	3.2	0.65	(0.61–0.69)
Use mammography within 365 days	87,944	2977	3.4	0.65	(0.63–0.68)

CI, confidence interval; OR, odds ratio; *Adverse events included with 30 day in-hospital mortality, pneumonia, septicemia, acute renal failure, stroke, and acute myocardial infarction; †Adjusted for all covariates listed in Table 1.

## Supplementary Materials

Supplementary materials can be found at https://www.mdpi.com/1660-4601/16/22/4432/s1. Table S1.

## Abbreviations

ICD-9-CM	International Code of Diseases, Ninth Edition, Clinical Modification
OR	odds ratio
CI	confidence interval
