# Supplementary material for: Previous Use of Mammography as a Proxy for General Health Checks in Association with Better Outcomes after Major Surgeries
Source: Int J Environ Res Public Health. 2019 Nov 12;16(22):4432. doi: 10.3390/ijerph16224432 (PMC6888288; doi:10.3390/ijerph16224432)
Supplement: Supplementary file 1 [file ijerph-16-04432-s001.pdf]

Table S1. Characteristics of surgical patients with and without preoperative use of mammography (before matching).

	Use of mammography				p-value
	No (N=504102)		Yes (N=194206)		
**Age, years**	**n**	**(%)**	n	(%)	<0.0001
47-49	75684	(15.0)	21315	(11.0)	
50-54	112057	(22.2)	47081	(24.2)	
55-59	109327	(21.7)	47304	(24.4)	
60-64	94590	(18.8)	42019	(21.6)	
65-70	112444	(22.3)	36487	(18.8)	
Low income					<0.0001
No	495010	(98.2)	191528	(98.6)	
Yes	9092	(1.8)	2678	(1.4)	
Number of hospitalizations					<0.0001
0	368585	(73.1)	147237	(75.8)	
1	86311	(17.1)	32560	(16.8)	
2	24771	(4.9)	8032	(4.1)	
≥3	24435	(4.9)	6377	(3.3)	
Number of emergency visits					<0.0001
0	326385	(64.8)	131716	(67.8)	
1	105826	(21.0)	39097	(20.1)	
2	38798	(7.7)	13432	(6.9)	
≥3	33093	(6.6)	9961	(5.1)	
Types of surgery					<0.0001
Musculoskeletal	171759	(34.1)	61461	(31.7)	
Digestive	82025	(16.3)	29684	(15.3)	
Neurosurgery	63552	(12.6)	24985	(12.9)	
Kidney, ureter, bladder	34740	(6.9)	13512	(7.0)	
Respiratory	18090	(3.6)	7976	(4.1)	
Cardiovascular	15201	(3.0)	4218	(2.2)	
Eye	7723	(1.5)	2338	(1.2)	
Skin	8064	(1.6)	2315	(1.2)	
Delivery, CS, abortion	935	(0.2)	360	(0.2)	
Others	102013	(20.2)	47357	(24.4)	
Types of anesthesia					<0.0001
General	406738	(80.7)	159221	(82.0)	
Epidural or Spinal	97364	(19.3)	34985	(18.0)	
Medical conditions					
Hypertension	142383	(28.2)	56751	(29.2)	<0.0001
Mental disorders	92189	(18.3)	40085	(20.6)	<0.0001
Cancer	72050	(14.3)	30755	(15.8)	<0.0001
Diabetes	84117	(16.7)	29972	(15.4)	<0.0001
COPD	41915	(8.3)	19605	(10.1)	<0.0001
Ischemic heart disease	37533	(7.5)	16217	(8.4)	<0.0001
Atherosclerosis	14951	(3.0)	6022	(3.1)	0.0031
Liver cirrhosis	11852	(2.4)	4857	(2.5)	0.0002
Heart failure	8944	(1.8)	2576	(1.3)	<0.0001
Stroke	14268	(2.8)	3194	(1.6)	<0.0001
Renal dialysis	14164	(2.8)	1911	(1.0)	<0.0001
Parkinson's disease	4131	(0.8)	1217	(0.6)	<0.0001

COPD, chronic obstructive pulmonary disease
